# Combined analytical approach empowers precise spectroscopic interpretation of subcellular components of pancreatic cancer cells

**DOI:** 10.1007/s00216-023-04997-w

**Published:** 2023-10-31

**Authors:** Krzysztof Szymoński, Katarzyna Skirlińska-Nosek, Ewelina Lipiec, Kamila Sofińska, Michał Czaja, Natalia Wilkosz, Matylda Krupa, Filip Wanat, Magdalena Ulatowska-Białas, Dariusz Adamek

**Affiliations:** 1 Department of Pathomorphology, Medical College, Jagiellonian University, Kraków, Poland; 2 Department of Pathomorphology, University Hospital, Kraków, Poland; 3 Faculty of Physics, Astronomy and Applied Computer Science, M. Smoluchowski Institute of Physics, Jagiellonian University, Kraków, Poland; 4 Doctoral School of Exact and Natural Sciences, Jagiellonian University, Kraków, Poland; 5 AGH University of Krakow, Faculty of Physics and Applied Computer Science, Kraków, Poland

**Keywords:** Pancreatic cancer, Ampullary cancer, Molecular spectroscopy, Convolutional neural networks, Raman imaging, Spectral analysis

## Abstract

The lack of specific and sensitive early diagnostic options for pancreatic cancer (PC) results in patients being largely diagnosed with late-stage disease, thus inoperable and burdened with high mortality. Molecular spectroscopic methodologies, such as Raman or infrared spectroscopies, show promise in becoming a leader in screening for early-stage cancer diseases, including PC. However, should such technology be introduced, the identification of differentiating spectral features between various cancer types is required. This would not be possible without the precise extraction of spectra without the contamination by necrosis, inflammation, desmoplasia, or extracellular fluids such as mucous that surround tumor cells. Moreover, an efficient methodology for their interpretation has not been well defined. In this study, we compared different methods of spectral analysis to find the best for investigating the biomolecular composition of PC cells cytoplasm and nuclei separately. Sixteen PC tissue samples of main PC subtypes (ductal adenocarcinoma, intraductal papillary mucinous carcinoma, and ampulla of Vater carcinoma) were collected with Raman hyperspectral mapping, resulting in 191,355 Raman spectra and analyzed with comparative methodologies, specifically, hierarchical cluster analysis, non-negative matrix factorization, T-distributed stochastic neighbor embedding, principal components analysis (PCA), and convolutional neural networks (CNN). As a result, we propose an innovative approach to spectra classification by CNN, combined with PCA for molecular characterization. The CNN-based spectra classification achieved over 98% successful validation rate. Subsequent analyses of spectral features revealed differences among PC subtypes and between the cytoplasm and nuclei of their cells. Our study establishes an optimal methodology for cancer tissue spectral data classification and interpretation that allows precise and cognitive studies of cancer cells and their subcellular components, without mixing the results with cancer-surrounding tissue. As a proof of concept, we describe findings that add to the spectroscopic understanding of PC.

**Graphical Abstract:**

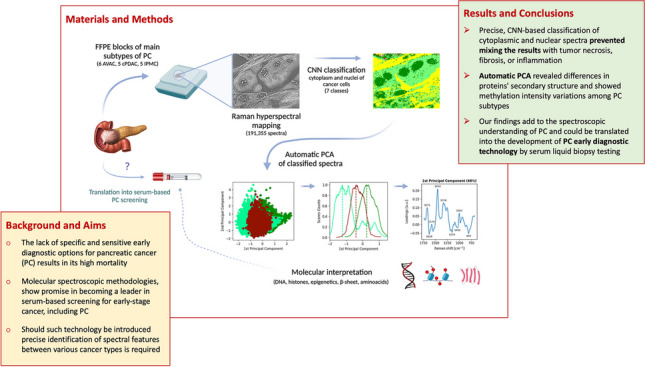

**Supplementary information:**

The online version contains supplementary material available at 10.1007/s00216-023-04997-w.

## Introduction

The rising understanding of pathomechanisms of pancreatic cancer (PC) initiation and evolution that we have witnessed in recent decades [1] has not improved the very low PC 5-year survival rates, which remain below 10% [2]. Due to the lack of specific and sensitive early diagnostic options, patients are largely diagnosed with late-stage disease [1]. The PC tumors’ molecular and morphological heterogeneity is also responsible for the PC being chemoresistant to available treatment options [1]. These are the main reasons for the drastically poor prognosis of PC patients [1]. New methods of studying the molecular composition of PC are required to develop efficient early detection technologies, as well as to extend the knowledge of the mechanisms of chemoresistance [3] and counteract them. Despite the urgent need for efficient and universal malignancy screening technologies, currently, none would fulfill the criteria of good specificity and sensitivity for PC. However, multiple serum-based biomarkers have been proposed without satisfactory results [4–6]. Some authors reported better usefulness in assessing interleukin-6 (IL-6) serum levels in differentiating PC patients from chronic or acute pancreatitis [7–9], or recently, leukemia inhibitory factor (LIF) was reported to be a promising serum biomarker of pancreatic malignancy [10]. Nevertheless, all these are only singular protein markers, which entails unsatisfactory diagnostic specificity and sensitivity [11]. Moreover, the single-biomarker methods cannot generalize to multiple varieties of cancer.

Serum liquid biopsy samples are considered ideal for cancer screening [6, 11]. Nevertheless, conventional liquid biopsy biomarkers (LBMs) comprising of circulating tumor nucleic acids (i.e., ctDNA, ctRNA), circulating tumor cells (CTCs), or extracellular vesicles (EVs) have not been introduced into medical practice because of limitations of the molecular/genetic testing techniques, such as described in [6].

A promising twist in the liquid biopsy analysis might be brought by the implementation of methods of vibrational spectroscopy (VS) such as surface-enhanced Raman spectroscopy (SERS) or attenuated total reflection Fourier-transformed infrared spectroscopy (ATR-FTIR) [3, 12]. VS was confirmed to be an excellent tool for the characterization of malignant tissue’s chemical structure and composition [13–16]. Due to the fingerprint-like character of resulting data acquired from VS, all information about the studied sample is ready for interpretation, making VS a universal technique of molecular characterization. Nevertheless, only a few studies utilized VS for the differentiation of multiple malignancies [17, 18], with most papers comparing only malignant *vs.* benign control [19]. Further exploration is needed to reveal the full potential of molecular spectroscopy in cancer screening. The only way for promising VS to be introduced as a diagnostic technology is by detailed characterization of the spectral results obtained for various cancer types.

Following this, in our study, we aimed to deepen the knowledge of VS landscapes of PC tumors by using an innovative and comprehensive methodology, including combined Raman hyperspectral mapping (RHM), conventional multivariate data analysis, and deep networking techniques. This approach allowed for the cognitive recognition of spectral markers of PC. We separately measured and analyzed cellular nuclei, the cytoplasm, and the tumors’ stroma compartment of main groups of PC, specifically the conventional pancreatic ductal adenocarcinoma (cPDAC), intraductal papillary mucinous carcinoma (IPMC), and ampulla of Vater adenocarcinoma (AVAC). Often these tumors are indistinguishable using standard histopathological evaluation techniques (i.e., the tumor’s epicenter location, morphology, and immunohistochemistry) [1, 20, 21]. Although current clinical management protocols recommend treating these tumors similarly, significant differences in cancer differentiation level, the occurrence of perineural and venous invasion, and lymph node involvement were reported [22]. A large study on cPDAC vs AVAC (476 vs 232 cases) resulted in showing significant differences in patients’ survival, specifically 15.6 vs 41 months, for cPDAC and AVAC, respectively [23]. Notably, in this study by Reid et al., the authors describe no impact of tumor size and lymph node metastasis on the patients’ survival [23].

Methods of VS complement each other and are usually used in different ways considering the type of samples that are to be studied. For example, as stated by some authors [3, 24, 25], tissues are best measured by the RHM technique, whereas blood serum probing involves highly sensitive SERS [26–30] and ATR-FTIR [31–34], which do not provide spatial resolution; however, they are effective in the investigation into bulk samples such as blood serum, thus ideal for early diagnostic of malignancies, such as PC. We believe that for the successful implementation of VS serum-based diagnostic technologies, firstly, an understanding of the spectroscopic characteristics of the tumors is required. Techniques of hyperspectral imaging (such as RHM) allow precise selection of cancer areas to further analyze them. The learned knowledge might be subsequently translated into serum-based liquid biopsy ATR-FTIR or SERS measurements.

Because of the nature of the Raman effect, VS is very sensitive to distortion factors. Fluorescence, thermal noise, and the measuring equipment quality might have a major impact on the results; thus, typically spectra preprocessing is required. Usually, it involves cosmic ray removal, baseline correction, and smoothing. During these operations, some seemingly irrelevant data might be lost if an improper preprocessing model was applied [35]. Conversely, CNNs are surprisingly efficient in classifying raw, unpreprocessed data [36], although they generalize better if unmeaningful information is removed. The successful use of neural networks, such as CNNs in spectroscopic data evaluation and classification, was shown in multiple studies [24, 36, 37]. Briefly, the CNN is trained by allowing it to analyze spectra from the so-called training dataset, from which CNN identifies characteristic features. The main advantage of CNN among other neural network types is its ability to self-extract discriminating features (automatic features extractor) [38]. Although manual feature extraction with proper setup may be as good as automatic in some classification scenarios [39], in cancer diagnostics, automation and high throughput of the process are crucially important [3].

RHM enables high-resolution imaging of tissue samples without the need for special labeling and with comparable costs to other standard techniques, such as magnetic resonance [40]. RHM is a molecular spectroscopy technique that utilizes multiple RS measurements of adjacent parts of the studied sample, followed by plotting the combined results as a tissue map image. The use of Raman spectroscopy (RS) to investigate cancer tissue samples is not new [14, 15]; however, only a few studies utilized RHM for the cognitive selection of areas of analysis [24, 25]. Contrary to RS random blind spot measurements [41] or rare grid mapping [42], this approach prevents mixing the results with areas of necrosis, inflammation, fibrosis, or colloid [3]. Without this precision, the interpretation of the molecular contents of PC cells is impossible, let alone the cellular cytoplasm and nuclei separately.

Here, as a first part of the study, we conducted comparative studies between spectral data analysis methodologies to reveal the fittest for defining spectroscopic landscapes of cytoplasm and nuclei of PC subgroups separately. Specifically, we compared hierarchical cluster analysis (HCA), non-negative matrix factorization (NMF), T-distributed stochastic neighbor embedding (tSNE), principal components analysis (PCA), and convolutional neural networks (CNN). Each has advantages and limitations, which we describe briefly in the *Supplementary section – Methods of multivariate data analysis used in the study*. In conclusion, we propose using a combined approach (CNN + PCA) that allows for automatic and high-throughput spectra classification and subsequent comprehensive characterization of the smallest spectral differences among them allowing molecular interpretation.

## Methods

### Tissue slide preparation

Raman imaging of 16 PC tissue slides from 15 patients was conducted. Specifically, 6 AVAC, 5 cPDAC, and 5 IPMC were included. The tissue samples were collected from patients with a diagnosis of PC who underwent pancreatoduodenectomy (Whipple or Traverso) or distal pancreatectomy, with the exclusion of benign pancreatic neoplasm or neuroendocrine neoplasm cases. The details of patients included in the study are summarized in Supplementary Table S1. In this study, we used methods of tissue slide preparation already described by others [24]. Briefly, tissue samples were selected from the Cracow University Hospital’s Pathomorphology Department’s archive, normally stored as conventional formalin-fixed paraffin-embedded (FFPE) blocks after the diagnostic process. The initial sample selection was performed by two independent experienced pancreatic pathologists, by assessing the standard hematoxylin–eosin-stained glass slides (H&E). A routine light microscope (Olympus BX53 Microscope, RRID:SCR_022568) was used for this stage. During the selection process, a detailed reevaluation of the tumor type was conducted, and initial diagnoses were confirmed. Subsequently, before Raman measurements, for each selected case, a single 2.5-μm-thick tissue section was sliced with a Microm® HM355S Automatic Microtome and mounted onto a CaF_2_ window (Raman Grade Calcium Fluoride substrates – CRYSTRAN LTD, England). Then, on unstained CaF_2_ slides, areas of cancer were marked by pathologists. A complete paraffin removal procedure was conducted involving a 12-h xylene bath and graded ethanol rehydration.

### Raman measurements

After PC tissue slide preparation, Raman measurements were executed using the already described procedure [24, 25]. Briefly, RS was conducted with a Horiba LabRam spectrometer equipped with a green (532 nm) laser and electron-multiplying charge-coupled device (EM-CCD) camera cooled to − 70 °C. During the measurements, a × 60 water immersion objective lens (Nikon) was used to allow measurements of tissue sections immersed in a physiological saline solution. Spectra were acquired in the fingerprint spectral region (1900–600 cm^−1^) with a spectral resolution of 2 cm^−1^. The RHM maps included 6724 to 13,284 spectra for a single slide in this study. The exposure time for each pixel was 6 s. The pixel size (step size) was 1 µm or smaller depending on the size of the preselected area of cancer (see “Tissue slide preparation”), which varied from 80 × 80 µm to 140 × 140 µm.

### CNN dataset annotation and data augmentation

Spectral data for the training of the CNN were selected from obtained RHM maps, by direct comparison with the unstained optical microscopy tissue slide images, normally collected before RS measurements. It was performed by the same pathologists, who preselected the areas of cancer (see “Tissue slide preparation”). Additional comparisons with H&E-stained slides were also performed, which clarified cellular components (such as the cancer cells’ nuclei) better. However, direct transmission of areas of nuclei and cytoplasm from H&E images was not possible, because the H&E slides were sliced from FFPE blocks before the tissues for RHM measurements, thus differing slightly from the investigated tissues. The LassoSelector widget from the MatPlotLib library (MatPlotLib, RRID:SCR_008624) of Python (IPython, RRID:SCR_001658) was used to annotate the data into 7 separate classes, including nuclear and cytoplasmic areas of each of the PC types (AVAC, IPMC, and cPDAC). Additionally, the stroma/empty class was annotated. Only a small part of all spectra in the RHM map was annotated for the training dataset (42,098 spectra from 191,355 — approximately 22%), leaving the rest for the CNN model validation with new data, testing its ability to generalize. The process of training dataset annotation is depicted in Supplementary Figure S1. Before feeding the data into CNN, it was preprocessed, which involved sequentially applying a baseline correction (3rd polynomial order), smoothing with the Savitzky–Golay algorithm (third-order, 15 smoothing points), and trimming in the spectral range characteristic for biological molecules (1800–650 cm^−1^). Subsequently, to prevent overfitting with the smoothed spectra, we applied data augmentation, by adding random noise to each spectrum. After the augmentation, the CNN training dataset included 126,294 spectra.

Additionally, to prevent the impact of different Raman shifts on the prediction results, spectral intensity values were packed with the Raman shift values into a single integer (int64) value.

After achieving 92% validation accuracy in the CNN training, to increase the model performance, we used another neural network dense classifier. This model required another training on different data. For each spectrum in the training dataset of the initial CNN model, we counted spectral ratios of (i) DNA methylation, (ii) β-sheet proteins, and (iii) random coil proteins. Some of these ratios were described to be specific for PC subtypes in VS spectra [24]. Specifically, for each spectrum, the relation between Raman bands characteristic of DNA methylation (δ(CH_2_, CH_3_), 1420–1360 cm^−1^ to ν_s_(PO_2_^−^), 1150–1050 cm^−1^), the β-sheet secondary structure of proteins (the β-sheet amide III, 1228–1218 cm^−1^ to total amide I, 1750–1514 cm^−1^), and proteins’ random coil secondary structure (the random coil amide I, 1640–1664 cm^−1^ to total amide I, 1750–1514 cm^−1^) were established. The counted ratios were combined with the spectral prediction result (performed by the initial CNN model) and fed into the shallow dense classifier.

### CNN architectures, training, and validation

In this study, we used two neural network models. First, for the spectra classification, a CNN was used, while for the final, combined spectral + ratio classification we utilized a shallow dense classifier. Below are the details of each of them.

The CNN was trained in predicting seven classes, representing separately the nucleus and the cytoplasm of AVAC, IPMC, and cPDAC, and additionally the stroma/empty space class. We used a custom-designed CNN architecture with 13 1D convolutional layers for feature identification and 3 fully connected layers for classification. A “sequential” base model was used. For each layer, a “glorot uniform” initializing mode was used. The “Adam” optimizer and “categorical crossentropy” loss function was applied. The CNN training involved 40 epochs with a batch size equal to 105.

The second dense classifier involved 3 fully connected layers. The batch size equaled 9 and the training took 17 epochs.

The total number of spectra used for CNN training was 126,294 with the 2D NumPy (NumPy, RRID:SCR_008633) array shape presented as (126,294, 320) and included spectra for each of 7 classes. Then, class arrays were “one-hot encoded,” and the training and testing dataset split was conducted with a 70/30 ratio. The initial CNN training time was 40 min.

Each 2D NumPy array of training dataset for the final classifier included values for (i) summed intensities of DNA bands, (ii) summed intensities of methylated DNA bands, (iii) DNA methylation ratio value, (iv) summed intensities of total proteins bands, (v) summed intensities of beta-sheet proteins bands, (vi) beta-sheet protein ratio, (vii) summed intensities of random coil protein bands, (viii) random coil protein ratio, and (ix) CNN prediction class for the spectrum. The shape of this NumPy array was (126,294, 9).

The programming of the CNN and dense classifier was performed in Python version 3.10.5 (IPython, RRID:SCR_001658) with TensorFlow (RRID:SCR_016345) and Keras application programming interfaces (API). The proposed CNN and dense classifier architecture details are summarized in Supplementary Figure S2.

After the CNN and final classifier were trained with satisfying performance, additional RHM spectral data was obtained from new PC patient tissues. This RHM map included 13,924 spectra not “seen” by the CNN during the training phase. The performance of the prediction on this tissue sample was recognized.

### Visualizing the CNN classification results

An efficient way of visualizing the spectral data prediction is to present the CNN classification results by plotting it as a tissue map image (prediction map) [24]. Every spectrum obtained with RHM for each tissue sample was fed into the CNN, followed by the final “ratios” classifier, and marked as one of the 7 classes. The predicted class values (classes 0–6 standing for stroma/empty, AVAC nucleus, AVAC cytoplasm, cPDAC nucleus, cPDAC cytoplasm, IPMC nucleus, and IPMC cytoplasm) created an array, which combined with the *x* and *y* coordinates of the original RHM map enabled the plotting of the prediction map. Each image pixel expressed a CNN-predicted class with a different color. The plotting was performed in Python (IPython, RRID:SCR_001658) with the MatPlotLib library (MatPlotLib, RRID:SCR_008624). The total number of spectra used for generating the prediction maps was 191,355, of which 149,257 were new spectral data not trained on by the CNN (approximately 78%).

### Multivariate data analysis methodology

Data analysis was conducted in the MATLAB (RRID:SCR_001622) environment from MathWorks (Natick, USA) (HCA, NMF) and in Python (IPython, RRID:SCR_001658) with Sklearn library (Sklearn, RRID:SCR_019053) (PCA, tSNE). Before the analyses, spectral data were preprocessed similarly to the spectra used for training the CNN (see “CNN dataset annotation and data augmentation”). The spectral range used for data analyses was trimmed to 1800–650 cm^−1^, which is desirable for biological structure examinations of samples obtained from FFPE tissue blocks. Certainly, in the higher spectral ranges (3,100–2,800 cm^−1^), one could expect the C-H stretching motions from methyl and methylene functional groups mainly from lipids; however, due to the required process of deparaffinization, the lipids in the samples were washed out (see “Tissue slide preparation”).

## Results

### CNN classifies Raman spectra of PC types with high accuracy and generalizes well on new data

**Fig. 1:**
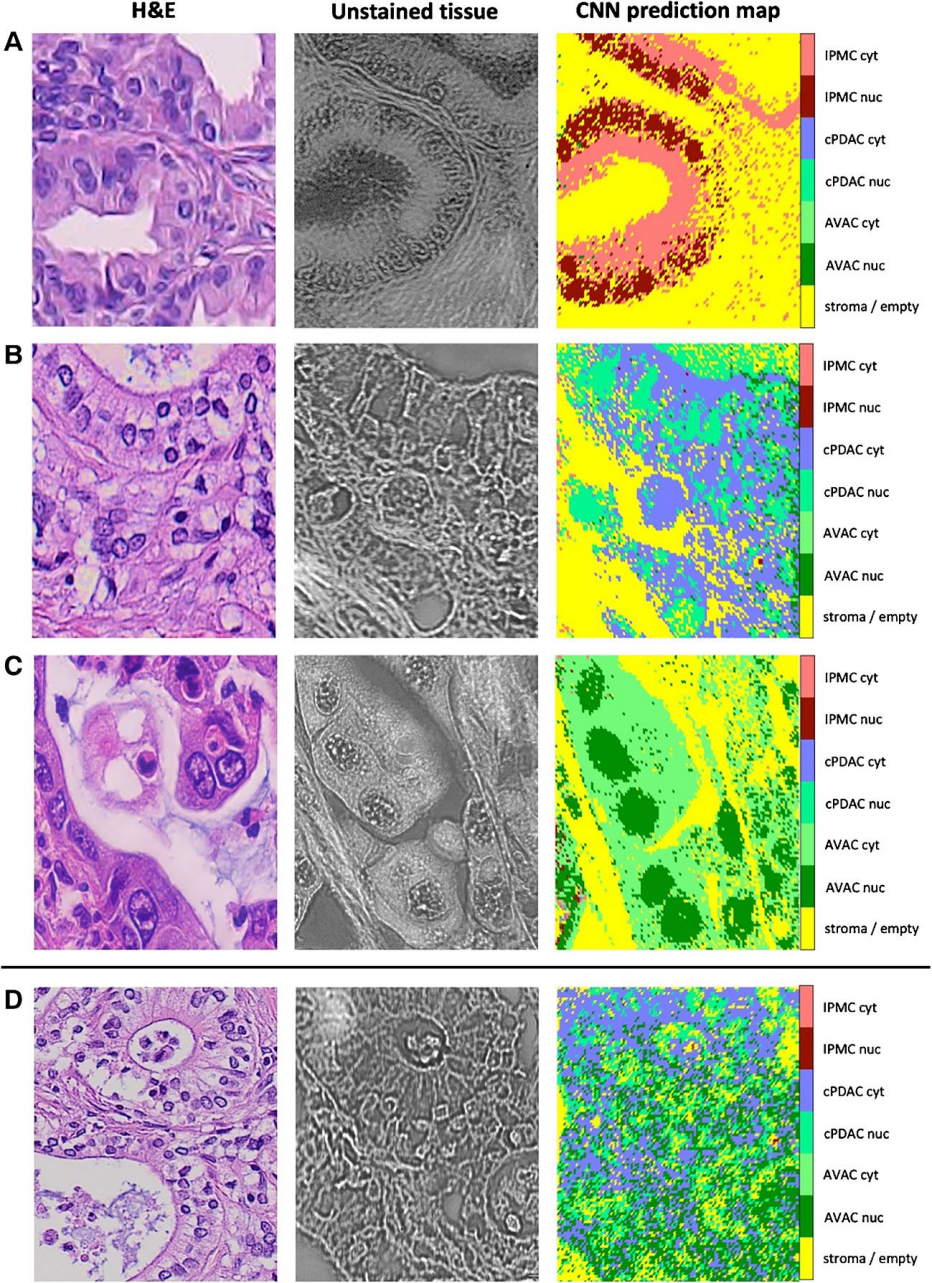
Tissue map images of PC tumors generated by the CNN. The samples of IPMC (**A**), cPDAC (**B**), and AVAC (**C**) tissues were classified by the CNN into 7 classes. Some of the spectra (about 22%) were initially annotated for the CNN training dataset. An additional RHM map was obtained from the cPDAC tissue section and fed into the pre-trained CNN (without retraining it with the new data) to check the generalization efficacy of the CNN model (**D**). Note that the H&E slides were sliced from the FFPE blocks before the slides used for the Raman measurements; thus, differences in tissue details might be observed (H&E, hematoxylin–eosin stain, original magnification 40×)

To retrieve the subcellular components of AVAC, cPDAC, and IPMC, we trained a custom CNN model (see “Methods” for a detailed description of the methodology). All spectra from RHM maps obtained from PC tissue sections were fed into pre-trained CNN in prediction mode. CNN classified the spectra into 7 classes (see “Visualizing the CNN classification results”). The validation accuracy of the prediction process reached slightly above 97%. The exemplary results of this part of our study are depicted in Fig. 1 as CNN prediction maps plotted for each type of PC tumor (AVAC, cPDAC, and IPMC). In that figure, the comparison of obtained map images to unstained light microscope tissue images, taken in the corresponding spot to the Raman measurements, proper cellular and subcellular tissue elements (the nuclei or cytoplasm) can be distinguished (classified adequately by the CNN). To the left of each figure 1st row panel, the H&E-stained tissue image is presented; however, these sections were sliced from the FFPE tissue blocks before the sections used for Raman measurements (see “Tissue slide preparation”); thus, they might be slightly different regarding the placement of tissue and cellular components. Nevertheless, the H&E image highlights the cells, nuclei, and cytoplasm of cells, showing a clear resemblance between unstained sections and CNN-predicted map images.

After achieving good CNN model validation performance on spectra obtained from the same PC tissues as these used for the training dataset (note that only approximately 22% of spectra were annotated, followed by training–validating dataset split in 70:30 ratio — see “CNN architectures, training, and validation”), to show the eligibility of our CNN model to generalize, we validated it on a new PC sample. To accomplish this, we sliced another tissue slide from the FFPE block obtained from a new PC case and conducted RHM measurements. None of the spectra from that tissue sample was used for CNN training, making the whole RHM map a validation field. The results of CNN prediction plotting are presented in Fig. 1D and show the proper classification of a very complex tissue section of cPDAC.

### Comparison studies of different spectral analysis methodologies

In Supplementary Figure S3, we present exemplary results of different analytical approaches to spectral data. The CNN (Supplementary Figure S3A) identifies spectra of interest (originating from such components as cellular nuclei and cytoplasm) with great accuracy (over 97%). Conversely, with HCA (Supplementary Figure S3B), the distinction of nuclear areas is less exposed. On the other hand, NMF reveals the distribution of separate molecular components, such as nucleic acids, proteins, or water (Supplementary Figures S3C, S3D, and S3E).

The results of PCA and tSNE were similar in our datasets (Supplementary Figure S4); however, tSNE would not provide insight into the spectral characteristics (such as the loadings plots for PCA) of PC subtypes’ cellular nuclei and cytoplasm, which was the main goal of our study — to recognize and describe the spectral landscapes of AVAC, cPDAC, and IPMC. As a result, we propose a combined approach, utilizing CNN for spectral classification of subcellular components and then PCA on the extracted spectra to identify the molecular details of each CNN-predicted class. This allows for an automatic, high-throughput, yet detailed characterization of PC tissue samples, on a subcellular scale. Moreover, this methodology might be adjusted in the analysis of any cancerous tissues.

After the successful CNN-based classification, spectra were analyzed by PCA concerning the distinction between those used for the CNN training dataset and the ones that the CNN model was validated on. In Supplementary Figure S3, the exemplary results considering spectra from the cytoplasm of AVAC cells are presented. As expected, such explorations revealed no significant spectral differences (Supplementary Figure S5A); however, the generalization ability of our CNN model is well illustrated with the extended plotting of the prediction spectra in the PCA scores (Supplementary Figure S5B).

### Variations in global DNA methylation and contents of β-sheet-rich intranuclear proteins among AVAC, cPDAC, and IPMC revealed with the PCA of Raman spectra CNN classified as PC nuclei

**Fig. 2:**
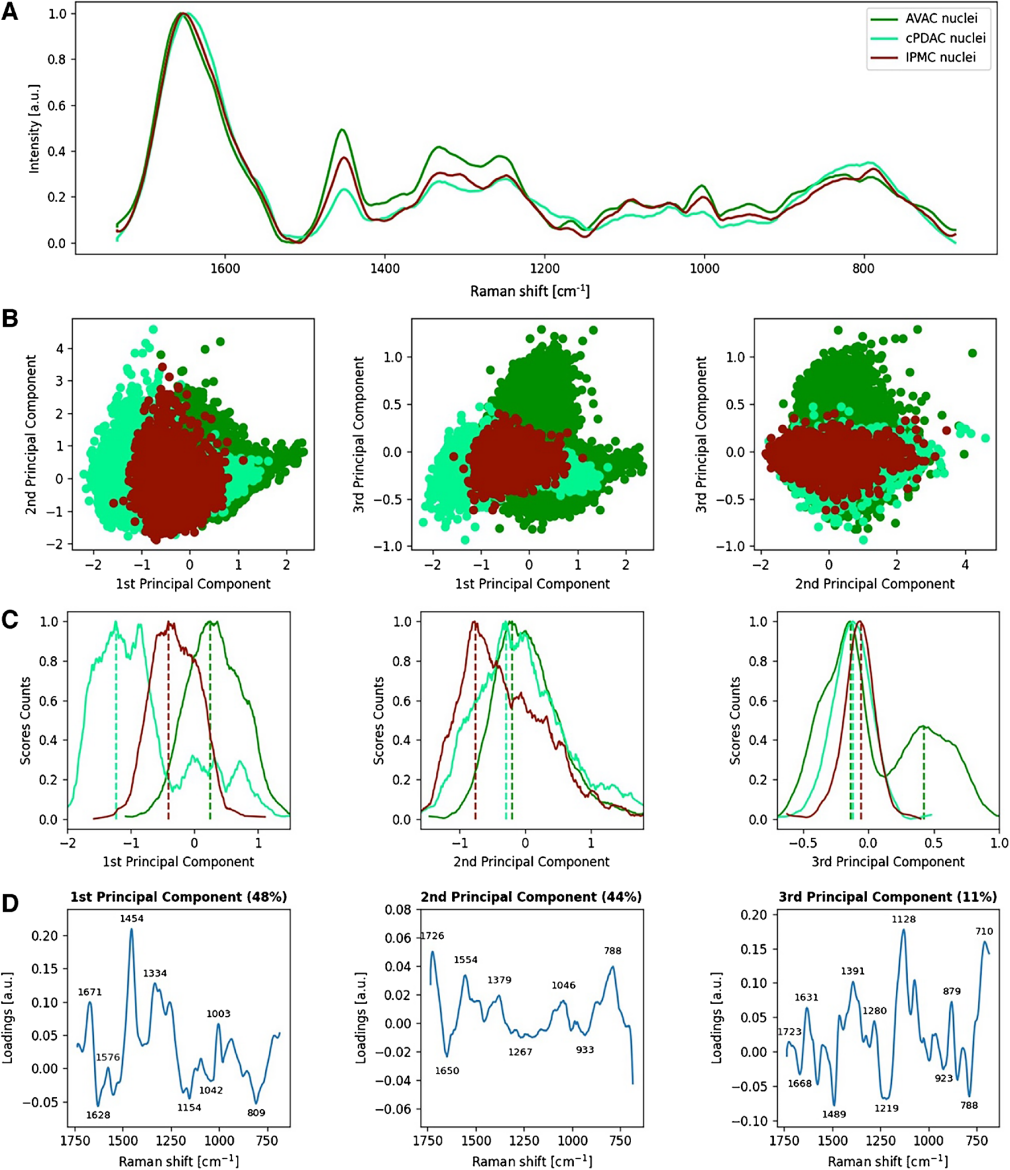
Molecular characteristics of the nuclei of PC tumors. Raman spectra from nuclei of all cells obtained from AVAC, cPDAC, and IPMC samples are presented as **A** linearly plotted mean spectra, **B** PCA 2D scores plots, **C** the PCA scores count plot showing the separation of their centroids, and **D** corresponding loadings plots with significant peaks marked

The results of PCA analysis performed on spectra extracted from nuclear areas of all studied PC types’ RHM maps are depicted in Fig. 2, as 2D score plots of spectra clustering (Fig. 2B) and the corresponding loading plots showing peaks responsible for spectra separation (Fig. 2D). For the sake of clarity, to show the centroid positions of PCA scores and their shifts along the principal components, the counts of the scores were plotted (Fig. 2C).

In Fig. 2, the loading plot, which explains separation along the principal component 1 (PC-1), is dominated by the protein bands such as the phenylalanine (Phe) ring breathing mode at 1004 cm^−1^; the amide I and amide III in the spectral ranges of 1700–1600 cm^−1^ and 1340–1230 cm^−1^, respectively; and the methyl and methylene bending motions at 1454 cm^−1^. All PC groups are clearly separated along PC-1, showing variable content of β-sheet secondary structure in nuclear proteins, the highest in cPDAC and the lowest in AVAC (amide I peak at 1628 cm^−1^). Spectra of cPDAC and IPMC nuclei are located on the negative side of PC-1, whereas almost all spectra acquired from AVAC nuclei are located peripherally at the positive side of PC-1. The corresponding maxima of PC-1 loadings are related to protein bands including the CH_2_ and CH_3_ bending, the amide bands, and the Phe ring breathing indicating a relatively high content of overall proteins in AVAC nuclei; however, combined with the above negative side of PC-1 interpretation, these proteins are less rich in β-sheet secondary structure than proteins in AVAV and cPDAC.

The principal component 2 (PC-2) explains 44% of the total variance within the considered dataset. The spectra acquired from IPMC nuclei are located at the negative side of PC-2, in contrast to those from cPDAC and AVAC nuclei, which are shifted towards the positive values of PC-2. The PC-2 loadings are negatively correlated with the already mentioned protein bands from the Phe, the amide III, and the CH_2_ and CH_3_ bending, indicating similarly to PC-1, the relatively high protein content in IPMC nuclei in comparison to cPDAC and AVAC. Moreover, a strong minimum of PC-2 is observed in the amide I spectral range at 1650 cm^−1^, showing the relatively high content of turns and unstructured coils secondary structures in nuclear proteins of IPMC.

The principal component 3 (PC-3), which explains 11% of the PCA total variance within the dataset, exhibits maxima related to the DNA backbone. These include 879 cm^−1^, 1070 cm^−1^, and 1128 cm^−1^, as well as from the methyl and methylene motions at 1489 cm^−1^, 1280 cm^−1^, and 1391 cm^−1^, indicating high methylation of DNA and/or histones. These bands are characteristic of spectra shifted towards positive values of PC-3, precisely a part of the spectra acquired from AVACs’ nuclei, revealing the local character of high methylation.

To summarize, PCA performed on spectra from the nuclei of PC cells differentiated AVAC, IPMC, and cPDAC. Although all PC types were rich in nuclear proteins, we found variable content of protein secondary structures among them. Specifically, β-sheet-rich proteins prevail in cPDAC, whereas turns and unstructured coils proteins are characteristic of IPMC nuclei. The high protein content in nuclei volume is considered a hallmark of the ongoing process of DNA repair [44]. Moreover, locally, in AVAC, a high DNA and/or histone methylation level was found; however, it was variable among PC types.

### The variable proteins’ secondary structure composition differentiates the cytoplasmic region of PC types determined by PCA of CNN-classified Raman spectra

**Fig. 3:**
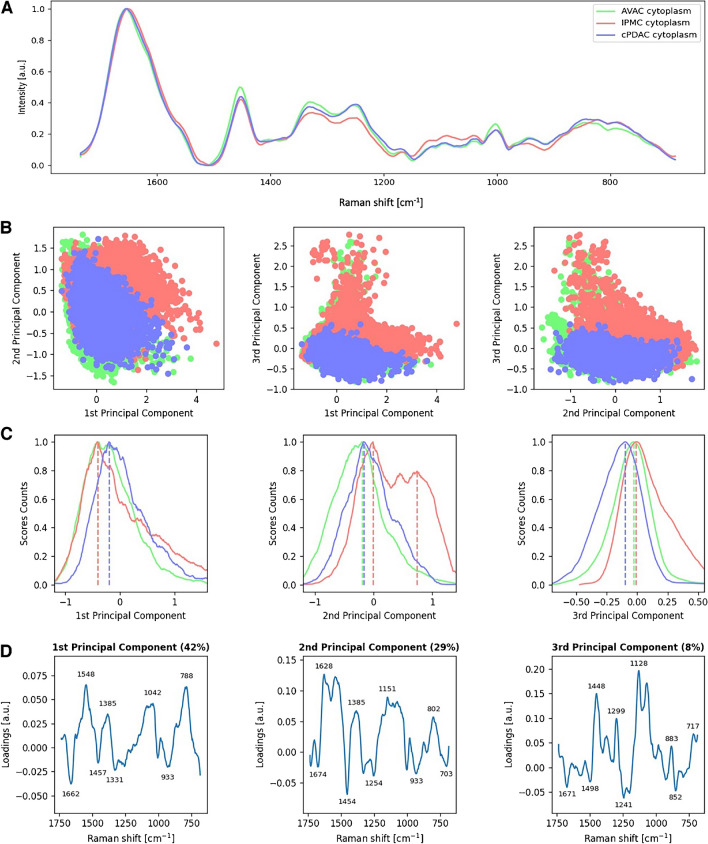
Molecular characteristics of the cytoplasm of PC tumors. Raman spectra from the cytoplasm of all cells obtained from AVAC, cPDAC, and IPMC samples are presented as **A** linearly plotted mean spectra, **B** PCA 2D scores plots, **C** the PCA scores count plot showing the separation of their centroids, and **D** corresponding loadings plots with significant peaks marked

Spectra acquired from the cytoplasm of IPMC, AVAC, and cPDAC exhibit grouping in the 3D space of PCA (Fig. 3B); however, a more discrete level of separation is observed, compared to the PCA of nuclear spectra. The separation along PC-1 (42% of the PCA total variance) is driven mainly by the minima related to protein bands including CH_2_, CH_3_ bending, the amide III, and the Phe ring breathing (Fig. 3D). Figure 3 B and C highlight these bands to be characteristic of IPMC, in contrast to cPDAC spectra, and thus, indicate a high content of cytoplasmatic proteins and peptides in cancerous cells of this type of PC tumors. Spectra of IPMC cells’ cytoplasm are shifted towards negative values of PC-1, and the characteristic minimum of PC-1 loading at 1662 cm^−1^ indicates a high contribution of turns and unstructured coils secondary structure in the proteins of IPMC cytoplasm.

There is only a slight separation of spectra acquired from the three investigated types of cancer along PC-2; however, the scores of IPMC are split into two groups, from which one is significantly shifted towards positive values of PC-2. The corresponding loading plot is dominated by maxima from proteins, including amide II at 1540 cm^−1^, the C–C, C-N, and C-O stretching modes in the spectral range from 1160 to 1050 cm^−1^, and C-H bending in tyrosine (Tyr) at 1170 cm^−1^ [45–48], suggesting local high content of Tyr-rich proteins in the cytoplasm of IPMC tumors.

PC-3 explains 8% of the PCA total variance and separates cytoplasm spectra of cPDAC (shifted towards lower PC-3 values) from AVAC and IPMC (PC-3 right-shifted). The PC-3 loadings are positively correlated with bands from the methyl and methylene motions from proteins and peptides at 1448 cm^−1^, 1299 cm^−1^, and 1378 cm^−1^, suggesting high content of methylated amino acids in the cytoplasm of AVAC and IPMC. Moreover, a strong positive correlation is also visible at 1128 cm^−1^, which was found to be a spectroscopic marker of reduced cytochrome C in mitochondria [49]. The negative correlation of PC-3 loadings, which is characteristic of spectra acquired from cytoplasmic areas of cPDAC, is observed for the band from the C-C_6_H_5_ stretching in Phe and tryptophan (Trp) at 1208 cm^−1^ indicating the presence of Phe- and Trp-rich proteins in cPDAC cells cytoplasm.

To summarize, the exploration of cytoplasmic parts of PC cells revealed a high content of turns and unstructured coil proteins in the IPMC, and what is more high content of Tyr-rich proteins was observed locally in the PMC cytoplasm. AVAC and some IPMCs were filled with methylated proteins, and a high content of reduced cytochrome C was detected. On the other hand, cPDAC cytoplasm included proteins rich in Phe and Trp.

## Discussion

It was shown that CNN efficiently differentiates between the main types of PC [24]. Currently, we deepen the insight into the PC’s molecular composition by extracting subcellular regions of cancerous tissues, allowing the distinction not only between AVAC, cPDAC, and IPMC but the nuclei and cytoplasm of their cells. This approach revealed new possibilities for investigating molecular characteristics of PC cells very precisely with considerable certainty of results interpretation. VS methods are great tools for molecular studies of cancerous tissues; however, the standard approach of random blind spot measurements [41] or rare grid mapping [42] might be prone to mixing the results with tumor necrosis and fibrosis, areas of inflammation, or benign pancreatic tissue. This leads to false results or misinterpretation. The VS methodology’s success depends on the cognitive selection of areas of measurement. The CNN-driven selection of target spectra is a very efficient, unsupervised, automatic, and high-throughput approach for errorless investigations into the nature of PC. The distinction between nuclear and cytoplasmic regions of cancer cells allows for separate interpretations of each, and thus, adds to the knowledge of the molecular nature of PC.

Various models of neural networks including CNNs were introduced to solve classification problems in medical science. Widely reported algorithms, which were successful in image recognition in pathology [50] or radiology [51–53], proteomics classification [54], or spectral data recognition for cancer diagnostics [19, 55–59], focus largely on classification only. Although CNNs are generally known to be efficient in predicting (true problem solvers), the mechanisms of that verdict are somehow hidden, making them unsuitable for studying the nature of the problem. Although CNNs are great at reading the slightest differences in spectral analysis, due to the “fingerprint” character of VS results, in the path of CNN prediction that information is lost, and only the classification result remains. The findings of our study show differently. We go beyond CNN-based classification, and we propose using CNN for spectral data biomolecular interpretation. Moreover, our approach is automatic, time-efficient, and might be unsupervised. We combined a very accurate CNN classifier with PCA which reduced the dimensionality of CNN-predicted spectra revealing significant differences in spectral bands characteristic of cellular nuclei and cytoplasm of main PC tumors, specifically AVAC, cPDAC, and IPMC. Both methods (CNN and PCA) utilize automatic feature extraction; however, using PCA only would not recognize the spectra of each class (in subcellular scale) with such specificity and accuracy as the CNN. Moreover, CNN has a great feature of generalization, meaning that it handles relatively well data that it has not been trained on before. On the other hand, CNN only would not highlight spectral variabilities between classes, making it useless in terms of molecular interpretation. Both methods supplement each other and together become a universal tool for molecular studies of tissue samples by VS (such as RHM).

Here, in an automatic manner, we identified cellular nuclei and cytoplasm regions of AVAC’s, cPDAC’s, and IPMC’s cells with a custom-designed CNN model. The annotation process for the CNN training required only less than 22% of the spectra to be marked, leaving the rest for the CNN generalization. First, we trained the CNN model by feeding it with raw spectral data. The achieved training and validation accuracy was very good (approximately 97%); however, that model did not perform well in predicting all seven classes when validated on new samples (new RHM map). To accentuate the meaningful information, spectra preprocessing were required. We removed the noise, baseline-corrected, and limited the range of analysis to that of biological samples (1800–650 cm^−1^). Not surprisingly, the CNN model trained well but relatively early it started to overfit (the validation accuracy would not improve above 85%). The data augmentation was performed, by adding random noise to the preprocessed spectra. Trained on this new dataset, our CNN model reached 92% in a successful validation. To further elevate the correct prediction score, we applied another neural network (NN) model — a shallow dense classifier. Some specific spectroscopic landmarks were recently established for PC subtypes, including global DNA methylation ratio or proteins secondary structure ratios [24]. These findings were included in generating the extended training dataset for the new NN, which involved combining the initial spectral CNN-based prediction class with calculated DNA methylation, proteins’ β-sheet, and proteins’ random coil secondary structure ratios. By using this, combined automatic (by CNN) and manual (by dense classifier) feature extractor approach, the final classification reached over 98%, and more importantly, it performed efficiently with the new dataset (new PC sample).

For the CNN analysis of data, it is often beneficial to take advantage of the CNN’s ability to extract spatial features. Although this type of analysis (called CNN-based image recognition) was largely applicated in medical sciences in radiology [60] or pathology [61], not many studies evaluated such an approach to spectral data analysis. When using RHM, it might be possible; however, we intentionally resigned from image recognition analysis, due to a couple of reasons. First of all, in our study, we aimed to recognize the molecular characteristics of PC tissues, for further direct translation of the results into other VS methodologies eligible for serum-based liquid biopsy testing. Among methods recognized as the most promising candidates in the diagnostics of malignancies including PC, the ATR-FTIR is leading the board [3, 59]. In this particular VS method, as a result of the measurement, a single infrared spectrum is generated. Thus, any spatial recognition, in that case, is not an option. Another reason why we resigned from studying spatial features in RHM map images was that it is hardly possible with good accuracy. To annotate the data for image recognition training of the CNN, one has to precisely select areas of a certain class and select them entirely. Although we could use for comparison the unstained tissue light microscope pictures, normally taken before the Raman measurements, with unstained and deparaffinized tissue, precise recognition of all cellular components (i.e., the nuclei and the cytoplasm) is impossible, especially when dealing with such complicated samples as PC tissues. The results of imprecise image recognition training might be a falsification. Last, but not least, the doubtful benefit of utilizing image recognition in our CNN model for the study results would be recognizable. Our approach of only spectral data CNN-based analysis was successful in 98% of spectra, the results of our studies can be directly translated into the development of PC diagnostic technology based on ATR-FTIR serum measurements, and we showed that the training of the CNN model with RHM data can be relatively easy, time-efficient, automatic, and have high throughput, thus showing the path for its implementation into studying of malignancies other than PC.

Subsequently, after successful classification, we proceeded to the characterization of each of the predicted classes, which included regions of nuclei and cytoplasm of AVAC, cPDAC, and IPMC. The automatic extraction of predicted spectra and the PCA of various pairwise and triple-wise combinations of classes were conducted, uncovering the differentiating spectral bands among them.

Analyses of both nuclear and cytoplasmic cellular regions of PC cells differentiated these main PC groups and allowed biochemical interpretations characteristic of each of them. Specifically, all PC samples were rich in nuclear proteins. This was an expected finding since a high nuclear protein content is considered a hallmark of the ongoing process of DNA repair [44]. Similarly, as expected among nuclear proteins, we found variabilities in their dominating secondary structure. Recently, the highest general content of β-sheet-rich proteins was found in the samples of AVAC [24]; however, in the current study, we found the nuclei of cPDAC to be dominated by β-sheet-rich proteins and IPMCs’ by turns and unstructured coils. To explain these discrepancies, it is worth noting that the NMF protein compound used for the analysis by the authors of the aforementioned study [24] represented mainly cytoplasmic and extracellular matrix proteins, while we investigated spectra selected from the nuclei of the PC cells, thus not contaminated with other cellular and extracellular regions of the studied tissue samples.

Both secondary structures we found (β-sheet, turns, and random coils) are domains of various proteins involved in carcinogenesis. In cellular nuclei, histones represent a substantial protein content, involved in DNA packaging or gene expression regulation [62]. Nevertheless, histones are primarily known for their α-helical structure. On the other hand, it was found that the β-sheet secondary structure promoted abnormal protein aggregation [63], a hallmark of cancer initiation and progression [64]. A great example of such protein is p53, which creates β-sheet aggregates in cellular nuclei of multiple cancers, including PC [65]. Another insulin-like growth factor-binding protein 2 (IGFBP2) was found to be associated with worse PC patients’ prognosis, by inducing the nuclear translocation and phosphorylation of the p65 subunit of nuclear factor kappa-light-chain enhancer of activated B cells (NF-κB) [66]. The latter (NF-κB) is a nuclear protein complex containing subunits rich in turns and random coils that are important for interacting with other proteins involved in transcriptional regulation [67]. Another random coil-rich protein is breast cancer type 1 susceptibility protein (BRCA1), which is critically involved in the homology-directed repair pathway (HDR) of double-strand breaks (DSB) of DNA [68] and was found to play an important role in PC tumorigenesis [69].

Aside from the protein structural variations, in the studies of nuclear spectra, we found high methylation rates of DNA and histones, most prevailing in AVAC and IPMC samples, which is in line with recent findings of DNA methylation among PC types [24]. DNA methylation and histone methylation are major players in epigenetic modification important in PC growth and progression [70]. The importance of a deeper understanding of these alterations is well presented by DNA methylation profiling-based classification of the central nervous system tumors [43]. The knowledge of epigenetic variabilities among PC types shows promise for the development of new targeted therapies [71].

The analyses of PC cell cytoplasmic regions revealed a high Tyr content in IPMC samples, whereas the proteins of cPDACs cytoplasm were abundant in Phe and Trp. Examples of Tyr-rich cytoplasmic proteins involved in PC development and progression are Src kinase and focal adhesion kinase (FAK). Both interact with each other and combined with other proteins’ deregulation; they drive tumor–stroma crosstalk and promote PC survival, adhesion, migration, and invasion [72]. Another Tyr-rich protein, STAT3, is constitutively activated in PC by the phosphorylation of Tyr705, leading to PC tumor progression at multiple stages of tumorigenesis, starting with the Pdx1 transcription factor-driven initiation of acinar-to-ductal metaplasia [73] (which is believed to be a precursor lesion of PC [1]). Yet, another Tyr-rich protein is phosphoinositide 3-kinase (PI3K), an important part of the PI3K/AKT/mTOR signaling pathway, which plays a key role in regulating PC cell growth and survival, and importantly might be targeted with PI3K inhibitors [74]. Additionally, the cytoplasmic proteins of some of the studied IPMC samples contained methyl and methylene-rich proteins. Hypermethylation of cytoplasmic proteins, such as the Ras association domain family 1A (RASSF1A) promoter, was found in 64% of PCs in the study by Dammann et al. [75]. This epigenetic event downregulates RASSF1A and lowers its action, such as stimulation of mitotic arrest, DNA repair, and apoptosis, thus inhibiting tumor suppressor activity [75].

An important finding was made in AVAC and IPMC samples, in which we detected a high content of reduced mitochondrial cytochrome C. The tyrosine phosphorylation of Tyr48 in mitochondrial cytochrome C puts it in the redox state, thus preventing cancer cell apoptosis [76] and driving cancer progression [77].

## Conclusions

The goal of our study was to extend the knowledge about the molecular nature of pancreatic tumors and find the best way of gaining it. These cancers are burdened with very high mortality, despite nearly daily reports adding to their understanding, multiple ongoing new therapeutic trials, or introduction of early diagnostic attempts. In serum-based liquid biopsy testing, as some authors [3], we perceive a solution to elevating the survival rates of PC patients; however, the conventional LBMs have not been introduced into clinical practice, because of the complexity of the methodology, and their not satisfying specificity, although comparable with other single-marker techniques [6]. Blood serum testing by VS combined with neural networking (including CNN) was established to be efficient in differentiating patients with and without malignancies [19, 55–59]. Nevertheless, for successful diagnostic technology, the ability to discriminate various malignancies in a single serum test is crucial; otherwise, one cannot be sure if the detected malignancy is truly the PC or maybe a gastric or colon cancer, making the test not useful in early screening for PC. A comparative study of various malignancies is required. Recently, in [24], the authors showed significant spectral variations among differentiated main groups of PC regarding global DNA methylation and the secondary structure of proteins. The results of their analysis, as well as ours, focusing on further interpretation of subcellular components of PC, can be directly translated into the development of PC diagnostic technology, which will be specific to PC (not other malignancies). However, this could not be achieved without our initial step taken in this study of characterizing the PC tissues first. It is due to the cognitive visualization by RHM and CNN prediction plotting, followed by PCA of each class that the true (not assumed) spectral interpretation was possible. A natural next step in establishing PC early screening technology is implementing our results in liquid biopsy testing. Early screening of PC patients is crucial for lowering the percentage of PC treatment failure, as it leads to early-stage tumors, making them suitable for radical resection and longer survival of patients [2].

### Supplementary information

Below is the link to the electronic supplementary material.

Supplementary file1 (PDF 887 KB)

## Data Availability

All data obtained during the studies are available from the corresponding authors upon reasonable request. The source codes for the CNN training, CNN-based prediction with map plotting, as well as PCA and other plotting, are available in a public git repository under: https://bitbucket.org/howkuen/avac_cpdac_ipmc_cnn/src/master/

## Abbreviations

PC: Pancreatic cancer
RHM: Raman hyperspectral mapping
VS: Vibrational spectroscopy
RS: Raman spectroscopy
NMF: Non-negative matrix factorization
tSNE: T-distributed stochastic neighbor embedding
PCA: Principal component analysis
HCA: Hierarchical cluster analysis
CNN: Convolutional neural network
NN: Neural network
IPMN: Intraductal papillary mucinous neoplasm
PanIN: Pancreatic intraepithelial neoplasia
cPDAC: Conventional pancreatic ductal adenocarcinoma
AVAC: Adenocarcinoma of the ampulla of Vater
IPMC: Carcinoma derived from IPMN
FTIR: Fourier transform infrared spectroscopy
ATR-FTIR: Attenuated total reflection Fourier transform infrared spectroscopy
SERS: Surface-enhanced Raman spectroscopy
Trp: Tryptophan
Tyr: Tyrosine
Phe: Phenylalanine
PC-1, PC-2, PC-3: Principal components 1, 2, 3
RASSF1A: Ras association domain family 1A
PI3K: Phosphoinositide 3-kinase
FAK: Focal adhesion kinase
HDR: Homology-directed repair pathway
DSB: Double-strand breaks
BRCA1: Breast cancer type 1 susceptibility protein
NF-κB: Nuclear factor kappa-light chain enhancer of activated B cells
IGFBP2: Insulin-like growth factor-binding protein 2

## Declarations

### Source of biological material

Tissue samples involved in the study were selected from the Cracow University Hospital’s Pathomorphology Department’s archive, normally stored as conventional formalin-fixed paraffin-embedded (FFPE) blocks.
