# Supplementary material for: Combined analytical approach empowers precise spectroscopic interpretation of subcellular components of pancreatic cancer cells
Source: Anal Bioanal Chem. 2023 Oct 31;415(29-30):7281–95. doi: 10.1007/s00216-023-04997-w (PMC10684650; doi:10.1007/s00216-023-04997-w)
Supplement: Supplementary file 1 — Supplementary file1 (PDF 887 KB) [file 216_2023_4997_MOESM1_ESM.pdf]

Combined analytical approach empowers precise spectroscopic interpretation of subcellular components of pancreatic cancer cells

Krzysztof Szymoński [1,2 *], Katarzyna Skirlińska-Nosek [3,4], Ewelina Lipiec [3], Kamila Sofińska [3], Michał Czaja [3, 4], Natalia Wilkosz [3,5], Matylda Krupa [1], Filip Wanat [1], Magdalena Ulatowska-Białas [1,2], and Dariusz Adamek [1]

[1] Jagiellonian University, Medical College, Department of Pathomorphology, Cracow, Poland
[2] University Hospital, Department of Pathomorphology, Cracow, Poland
[3] Jagiellonian University, Faculty of Physics, Astronomy and Applied Computer Science, M. Smoluchowski Institute of Physics, Cracow, Poland
[4] Jagiellonian University, Doctoral School of Exact and Natural Sciences, Cracow, Poland
[5] AGH University of Krakow, Faculty of Physics and Applied Computer Science, Cracow, Poland

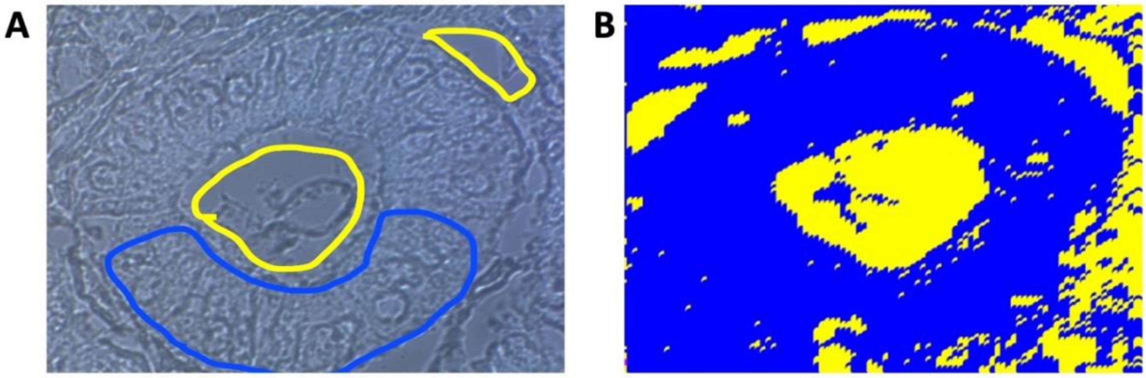

Supplementary Figure S1. The annotation process of CNN training dataset. On unstained tissue slides, a pathologist marked areas of cancer cells and the tumor stroma (or clean space) with different labels. The spectra from corresponding areas on the RHM map were extracted as the training dataset. As depicted in **(A)** only a fraction of the total areas was marked for each label (approximately 22% of spectra was extracted for the CNN training dataset), leaving the rest for the CNN as a validation dataset. The CNN model generalized well on the whole RHM map **(B)**, as confirmed by the pathologist.

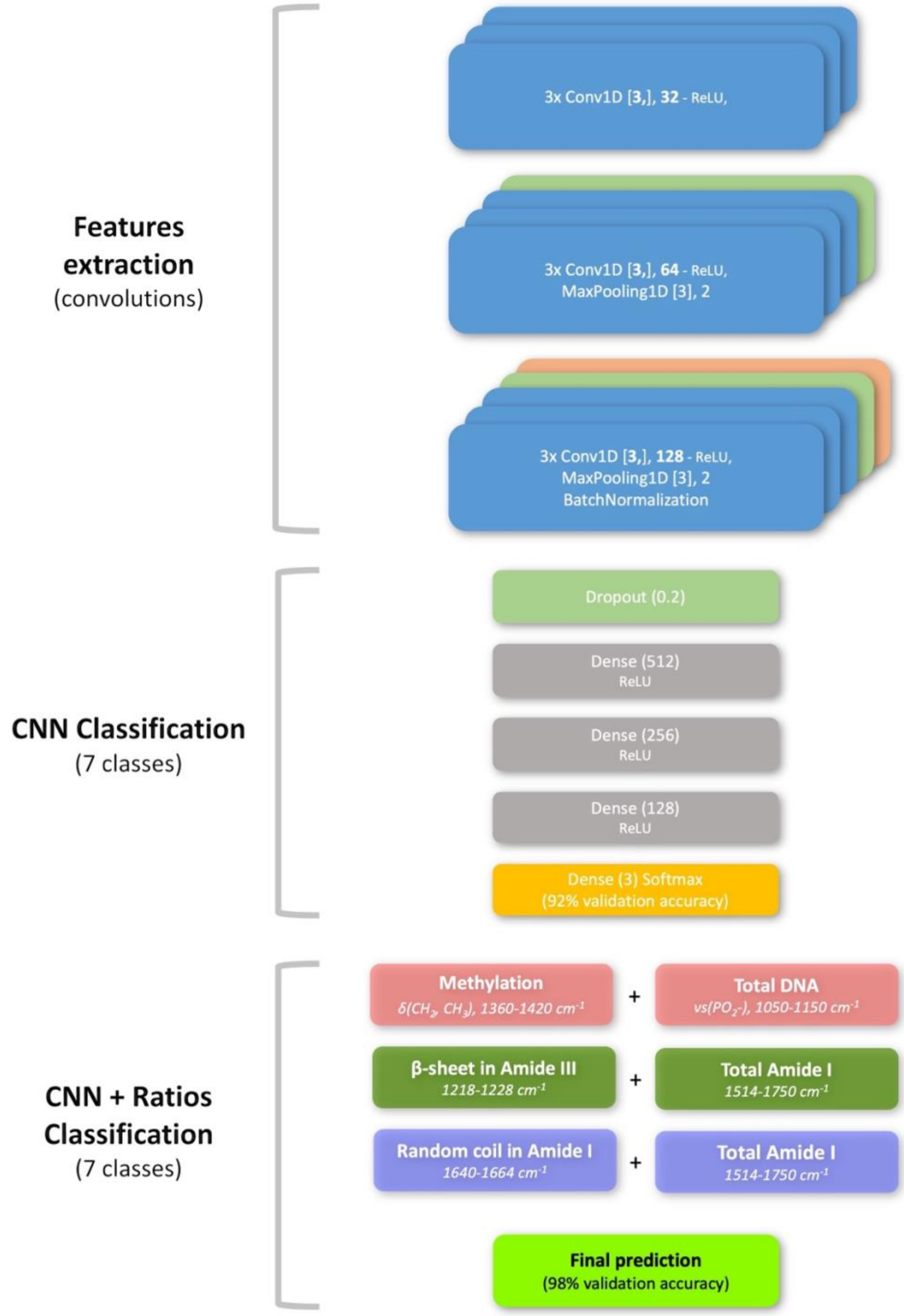

Supplementary Figure S2. Custom CNN and dense classifier architectures designed for training and testing in differentiating subcellular areas of pancreatic tumors (AVAC, cPDAC, and IPMC). The CNN was composed of a set of 3 convolutional layers with 32 filters (kernel size [3,]), followed by a set of 3 convolutional layers with 64 filters (kernel size [3,]) and max pooling (pool size [3,], stride [2]), and another set of 3 convolutional layers with 128 filters (kernel size [3,]), max pooling (pool size [3,], stride [2]), and a batch normalization layer. Then a dropout (0.2) layer and set of 4 fully connected classification layers. The ReLu activation was used for each convolutional layer. Afterward, another dense classifier was used that combined the CNN-based classification result with DNA methylation and proteins secondary structure ratios (see *Methods* section in the main document).

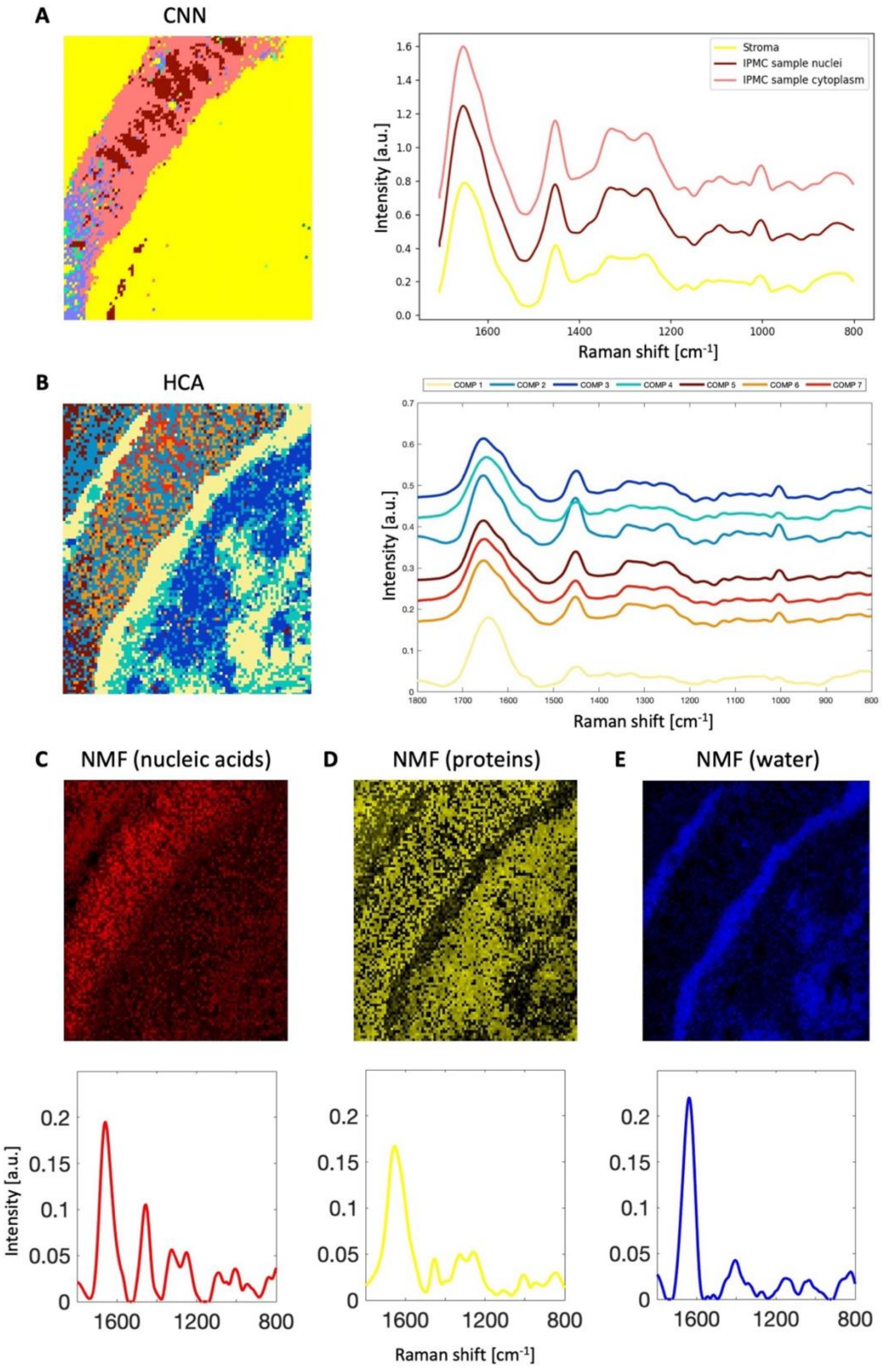

<u>Supplementary Figure S3. Various methods of PC tissues' spectral analysis.</u> CNN prediction map with mean Raman spectra of each class **(A)**, HCA map with mean Raman spectra of cluster components **(B)**, and NMF maps showing the distribution of nucleic acids **(C)**, proteins **(D)**, and water **(E)** with corresponding mean Raman spectra (below).

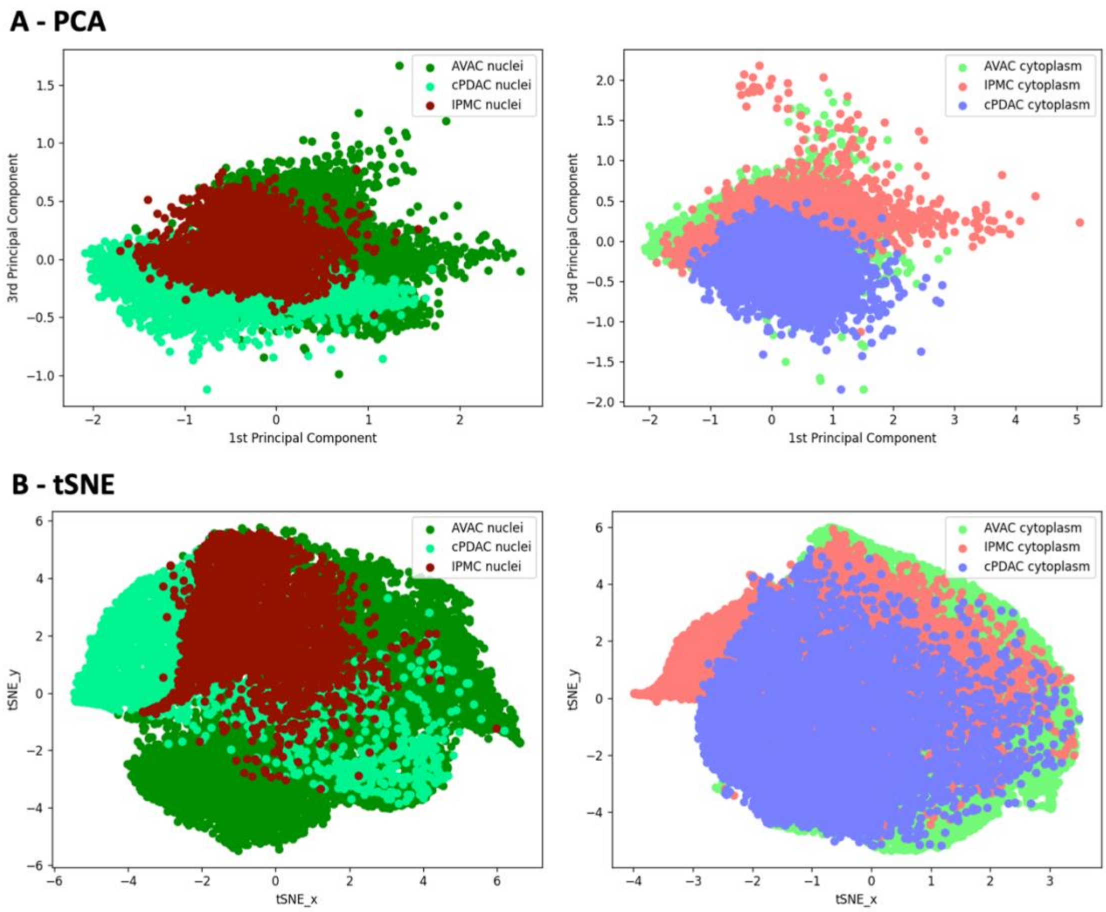

Supplementary Figure S4. The juxtaposition of high-dimensional data visualization techniques. The 2D scores plots obtained from PCA of Raman spectra classified by the CNN as PC subtypes' cellular nuclei (left) and cytoplasm (right) **(A)**, compared with the plotted results of tSNE of the same Raman spectra **(B)**.

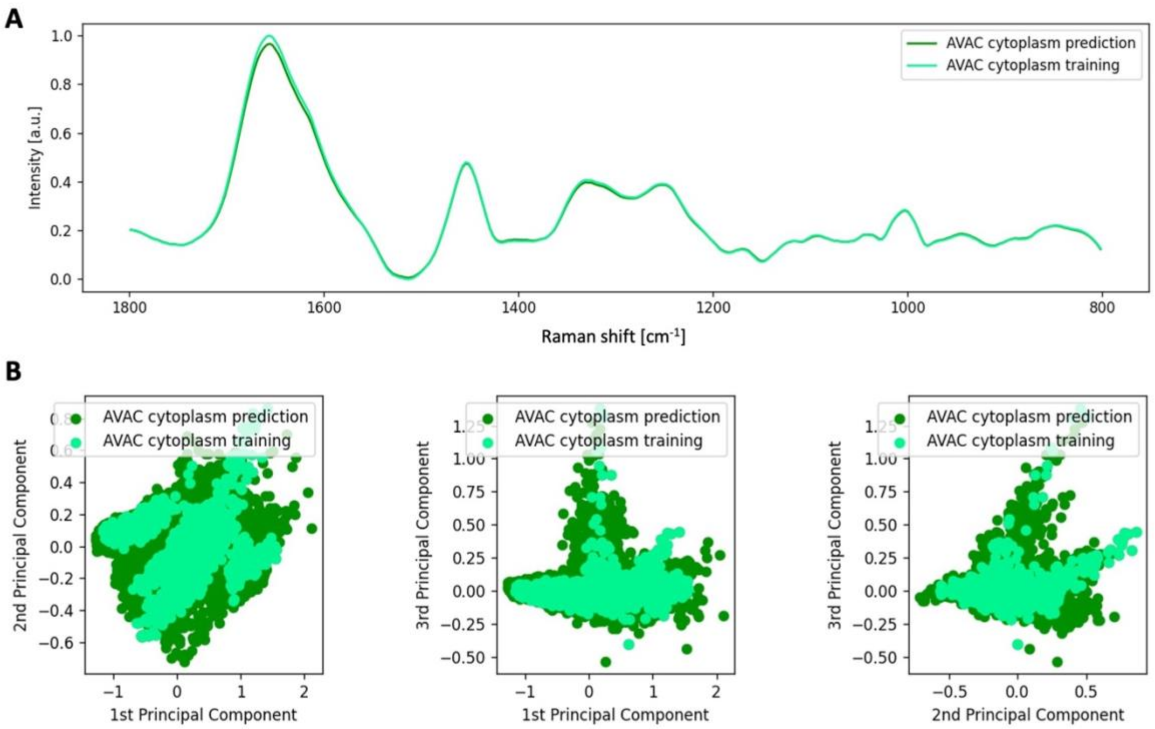

Supplementary Figure S5. CNN Prediction vs CNN Training. Exemplary comparison of the cytoplasm of all AVAC cells included in the CNN training dataset compared with the spectra classified by the pre-trained CNN as AVAC cytoplasm class depicted as **(A)** mean spectra – showing almost no spectral differences and **(B)** PCA 2D scores plots, which shows the overlapping of both groups. Moreover, the extended plotting of the prediction spectra highlights the generalization of the classification process.

Supplementary section – Methods of multivariate data analysis used in the study

We used and compared different multivariate spectral data analysis methods in this study. They all have advantages and disadvantages, some are better used in some specific conditions or expected results requirements. Below, these methods, including supervised and unsupervised techniques, are briefly described.

HCA is a useful method of clustering based on spectra similarity. As a result of the calculation, the algorithm produces dendrograms and allows for assembling the false-color maps. In this method, we observe the direct comparison, therefore the result is the mix of the signals from various bio-components [1]. Due to this limitation, the conclusions can be difficult to obtain as the interferential effect may be observed. However, with proper supervision and algorithm parameter adjustments, the resulting map images allow for the distinction of the subcellular components, such as the nucleus and cytoplasm [2].

In NMF the algorithm is based on decomposing the data matrix into two lower-rank non-negative matrices W of scores related to the individual chemical bases and H of factors. The main advantage of using the NMF approach is the possibility to identify and differentiate biochemical compounds (such as proteins, lipids, phospholipids, or nucleic acids) in the spectra with reasonable confidence based on the H matrix. Similar to the HCA method, the maps that present the occurrence region of the component are available after calculations. Importantly, NMF can extract the signal from water as one of the components; thus, revealing the initially "covered" signals from other components, such as proteins or nucleic acids). When analyzing the secondary structure of proteins this "uncovering" ability is important, otherwise, the O-H bending motion at 1643 cm^{-1} overlaps with the amide I band making the proper interpretation of proteins' structural conformation hard or impossible. Another solution to proteins' secondary structure interpretation might be analyzing the amide III region of the spectra (1228–1218 cm^{-1}) instead of amide I. Such an approach was utilized in the current study.

Although NMF is useful for analyzing whole RHM maps, and thus, good for tissue samples, the research performed on singular spectra, which the beforementioned, promising ATR-FTIR-based serum diagnostic results with, are more difficult. Because NMF extracts the general distribution of molecular components, such as proteins, by analyzing the whole RHM map, it lacks precision, so important in analyzing single cells in tissue samples. As depicted in Figures 2C, 2D, and 2E individual cells cannot be identified. Moreover, the whole map instead of a single spectrum interpretation, makes NMF prone to mixing the results with "unwanted" stromal cell compartments, such as inflammation cells (i.e., neutrophils, lymphocytes, or histiocytes) when analyzing nucleic acids NMF components (Figure 2C) or stromal extracellular proteins (i.e., extracellular matrix, collagen, and fibroblasts) when investigating proteins NMF components (Figure 2D).

An unsupervised tSNE is a common solution for "simplifying" and visualizing high-dimensional data. It has been widely used in the DNA methylation-based classification of tumors of the central nervous system [3]. It comprises complex math to reduce the number of dimensions in a non-linear fashion. In simple words, the algorithm embeds points from higher to lower dimensions, but without losing the neighboring points.

Another unsupervised technique, the PCA, comprises of automatic features extractor, however, it cannot classify. Instead, it produces multidimensional space plots (scores) based on the linear transformation of data to so-called principal components, which are described by orthogonal axes. The relation and separation of data can be recognized according to their influence on the scores. Additionally, PCA reveals the spectral differences among the data points by defining the "loadings", which highlight differentiating spectral bands.

Supplementary Table S1. Characteristics of patients included in the study. IPTN, Intraampullary papillary tubular neoplasm; IPMN, Intraductal papillary mucinous neoplasm; AJCC, American Joint Committee on Cancer staging classification 8th edition;

**#**	**Gender**	**Age**	**Tumor location**	**Diagnosis by WHO 5th edition**	**Grade**	**Stage** AJCC 8th
1	M	53	Ampulla of Vater	IPTN with an associated carcinoma intestinal type	G2	pT2
2	M	67	Common bile duct	Adenocarcinoma of common bile duct pancreatobiliary type	G2	pT3
3	F	73	Ampulla of Vater	IPTN with an associated carcinoma pancreatobiliary type	G2	pT1b
4	F	61	Pancreatic head	Ductal adenocarcinoma, NOS	G2	pT2
5	M	51	Pancratic tail	Ductal adenocarcinoma, NOS	G2	pT2
6	M	61	Pancreatic head	Ductal adenocarcinoma, NOS	G3	pT3
7	M	58	Pancreatic head	IPMN with an associated invasive carcinoma	G1	pT1a
8	M	76	Pancreatic head	IPMN with an associated invasive carcinoma	G2	pT3
9	F	68	Pancreatic head	IPMN with an associated invasive carcinoma	G2	pT2
10	F	83	Ampulla of Vater	Ampullary ductal carcinoma pancreatobiliary type	G2	pT3b
11	F	54	Ampulla of Vater	Ampullary ductal carcinoma pancreatobiliary type	G3	pT2
12	F	63	Pancreatic head	Ductal adenocarcinoma, NOS	G3	pT3
13	M	67	Pancreatic head	Ductal adenocarcinoma, NOS	G2	pT2
14	M	72	Pancratic tail	IPMN with an associated invasive carcinoma	G2	pT2
15	F	78	Pancreatic head	IPMN with an associated invasive carcinoma	G3	pT2

References

1. Kniggendorf A-K, Gaul TW, Meinhardt-Wollweber M (2011) Hierarchical Cluster Analysis (HCA) of Microorganisms: An Assessment of Algorithms for Resonance Raman Spectra. Appl Spectrosc 65:165–173. https://doi.org/10.1366/10-06064
2. Szymoński K, Lipiec E, Sofińska K, Skirlińska-Nosek K, Milian-Ciesielska K, Szpor J, Czaja M, Seweryn S, Wilkosz N, Birarda G, Piccirilli F, Vaccari L, Szymoński M (2021) Spectroscopic screening of pancreatic cancer. Clinical Spectroscopy 3:100016. https://doi.org/10.1016/j.clispe.2021.100016
3. Capper D, Jones DTW, Sill M, Hovestadt V, Schrimpf D, Sturm D, Koelsche C, Sahm F, Chavez L, Reuss DE, Kratz A, Wefers AK, Huang K, Pajtler KW, Schweizer L, Stichel D, Olar A, Engel NW, Lindenberg K, Harter PN, Braczynski AK, Plate KH, Dohmen H, Garvalov BK, Coras R, Hölsken A, Hewer E, Bewerunge-Hudler M, Schick M, Fischer R, Beschorner R, Schittenhelm J, Staszewski O, Wani K, Varlet P, Pages M, Temming P, Lohmann D, Selt F, Witt H, Milde T, Witt O, Aronica E, Giangaspero F, Rushing E, Scheurlen W, Geisenberger C, Rodriguez FJ, Becker A, Preusser M, Haberler C, Bjerkvig R, Cryan J, Farrell M, Deckert M, Hench J, Frank S, Serrano J, Kannan K, Tsirigos A, Brück W, Hofer S, Brehmer S, Seiz-Rosenhagen M, Hänggi D, Hans V, Rozsnoki S, Hansford JR, Kohlhof P, Kristensen BW, Lechner M, Lopes B, Mawrin C, Ketter R, Kulozik A, Khatib Z, Heppner F, Koch A, Jouvet A, Keohane C, Mühleisen H, Mueller W, Pohl U, Prinz M, Benner A, Zapatka M, Gottardo NG, Driever PH, Kramm CM, Müller HL, Rutkowski S, von Hoff K, Frühwald MC, Gnekow A, Fleischhack G, Tippelt S, Calaminus G, Monoranu C-M, Perry A, Jones C, Jacques TS, Radlwimmer B, Gessi M, Pietsch T, Schramm J, Schackert G, Westphal M, Reifenberger G, Wesseling P, Weller M, Collins VP, Blümcke I, Bendszus M, Debus J, Huang A, Jabado N, Northcott PA, Paulus W, Gajjar A, Robinson GW, Taylor MD, Jaunmuktane Z, Ryzhova M, Platten M, Unterberg A, Wick W, Karajannis MA, Mittelbronn M, Acker T, Hartmann C, Aldape K, Schüller U, Buslei R, Lichter P, Kool M, Herold-Mende C, Ellison DW, Hasselblatt M, Snuderl M, Brandner S, Korshunov A, von Deimling A, Pfister SM (2018) DNA methylation-based classification of central nervous system tumours. Nature 555:469–474. https://doi.org/10.1038/nature26000
